# Impact of sentinel lymph node assessment on the outcomes of patients with advanced endometrial cancer: A meta-analysis

**DOI:** 10.1097/MD.0000000000033465

**Published:** 2023-04-14

**Authors:** Huiyi Yao, Ruiwen Luo, Ruoyi Tong, Yanwen Wei, Kaiteng Zheng, Xiangdan Hu

**Affiliations:** a The Second Clinical College of Guangzhou University of Chinese Medicine, Guangzhou, Guangdong, China; b Department of Gynecology, The Second Affiliated Hospital of Guangzhou University of Chinese Medicine, Guangzhou, Guangdong, China.

**Keywords:** endometrial cancer, lymphadenectomy, sentinel lymph node

## Abstract

Sentinel lymph node (SLN) assessment is used to treat early endometrial cancer; however, its application in advanced endometrial cancer remains controversial. Therefore, we assessed the effects of SLN assessment and lymphadenectomy (LND) on the prognosis (survival rate and primary outcome) of patients with advanced endometrial cancer.

**Methods:**

Pertinent studies were selected from PubMed, Embase, Web of Science, and the Cochrane Library until March 19, 2022. Relevant studies were strictly screened according to the inclusion and exclusion criteria. Data from the included studies were extracted and their quality was evaluated. Then RevMan5.4 software was used for the meta-analysis.

**Results:**

Four retrospective studies were included, which enrolled 7181 patients; 492 were treated with SLN and 6689 with LND. In terms of overall survival, there was no significant difference between the 2 groups (odds ratio = 1.14, 95% confidence interval: 0.92–1.41, *I*^2^ = 0%, *P* = .39).

**Conclusions:**

SLN assessment is an alternative to LND as a treatment modality for advanced endometrial cancer.

## 1. Introduction

Endometrial cancer is the sixth most common cancer in women, with 41,700 newly diagnosed cases and 97,000 deaths worldwide in 2020.^[1]^ Owing to the prevalence of obesity and an aging population, the incidence of endometrial cancer is rising globally.^[2]^ Obesity is an independent risk factor; some studies have shown that being overweight or obese is related to high morbidity and, due to increased complications, may lead to a higher mortality rate.^[3,4]^ Although the incidence of endometrial cancer is increasing, most patients are diagnosed relatively early, and surgical treatment can have a good prognosis.^[5]^ Simultaneously, with effective individualized adjuvant therapy, the side effects and risks caused by overtreatment can be avoided. With the deepening of research, an increasing number of treatment methods are being developed and are progressing, such as immunotherapy and targeted therapy, which provide more survival opportunities for patients with malignant tumors.

Surgery is the mainstay treatment for endometrial cancer; the recommendation is comprehensive staging surgery, including abdominal exploration, pelvic peritoneal cytology, hysterectomy with bilateral salpingo-oophorectomy, and pelvic lymphadenectomy (LND) with or without para-aortic LND. The assessment of lymph node status is important to guide decisions regarding adjuvant therapy and inform on the prognosis, which is a central step in surgical staging.

Routine LND prolongs the surgery time and hospitalization, and increases the risk of a series of postoperative complications, such as vascular and nerve injury, lymphatic cysts, and lymphedema,^[6]^ which negatively affect patients’ quality of life. Some studies have found that LND does not significantly improve survival outcomes in patients with endometrial cancer because of the increased risk of postoperative complications.^[7,8]^ As a more targeted alternative, the sentinel lymph nodes (SLNs) fill the gap between lymph node staging and systemic lymph node resection. SLNs can be used to evaluate lymph node metastasis, providing a reference for subsequent treatment strategies. At the same time, SLN assessment can reduce the operation time and minimize collateral damage and postoperative complications^[9]^; it has also been included in the National Comprehensive Cancer Network guidelines as a valuable treatment option for the surgical staging of low-risk endometrial cancer^[10,11]^; however, its role in high-risk cases remains controversial.

Traditionally, the surgical approach for advanced endometrial cancer has been cytoreductive surgery, which includes the removal of all lymphoid tissue associated with the area. However, the role of LND for endometrial cancer remains controversial. As SLNs have become more widely used, their use in patients with endometrial cancer and extrauterine involvement is increasing.^[12]^ Moreover, several studies have found that the type of nodal assessment has no significant effect on survival outcomes in advanced endometrial cancer; therefore, comprehensive LND with SLN assessment has been proposed as an alternative treatment for advanced endometrial cancer. The main purpose of the present study was to compare the efficacy of different types of lymph node assessments, namely SLN assessment versus LND, on the prognosis of advanced endometrial cancer.

## 2. Materials and methods

### 2.1. Search strategy

A comprehensive search of PubMed, Embase, Wed of Science, and the Cochrane Library was limited to high-quality studies published until March 19, 2022. Relevant literature was searched to find studies that met the inclusion criteria, which included studies on the impact of SLN biopsy and LND on the prognosis of patients with endometrial cancer, including SLN assessment, endometrial neoplasms, and SLN biopsy.

### 2.2. Inclusion and exclusion criteria

The inclusion criteria were as follows: study type: studies comparing the prognosis of patients with advanced endometrial cancer between SLN and LND patients; the research subjects were patients with endometrial cancer diagnosed by histopathology before surgery, and their preoperative staging was International Federation of Gynecology and Obstetrics stage III to IV; the language was limited to Chinese and English; outcome indicators: survival rate, chemotherapy rates, radiotherapy rates, lymphatic invasion; original research report; and rigorous experimental design and reliable data. The exclusion criteria were as follows: the literature was a study published in the form of a review, case report, or conference abstract; the original literature was a non-controlled study (no control group); the required data could not be extracted or the full text could not be obtained; the sample size was too small (<10) or the sample inclusion criteria were not clear; suspected duplicate studies were excluded; non-single endometrial cancer, that is, studies with other malignant tumors at the same time; and outcomes: the study does not assess any of the above indicators.

### 2.3. Quality evaluation

The Newcastle–Ottawa Scale was used to evaluate the quality of the included studies, and a score of 6 to 8 indicated good quality.

### 2.4. Data extraction

Literature screening and data extraction were independently completed and crosschecked by 2 researchers. If there was any disagreement, a third party was consulted to assist with the judgment. First, we read the titles and abstracts for preliminary screening, and after excluding duplicate studies and those that obviously did not meet the inclusion criteria, we read the full texts of the literature that met the criteria for re-screening and finally determined whether to include them. Data extraction was carried out for the included literature, and the specific contents included general information such as research title, first author, publication year, and research year, and basic information such as sample size, grouping, research objects, and outcome indicators.

### 2.5. Statistical analysis

All calculations were performed using the statistical software provided by the Cochrane Collaboration (RevMan 5.4). The combined effect size selected the 95% confidence interval (CI) and odds ratio (OR) as the effect indicators of the study, and the *P* value was obtained by Z test, and *P* < .05 was considered to be statistically significant. Heterogeneity was assessed based on the inconsistency of *I*^2^ measurements. If there was heterogeneity (*P* < .1, *I*^2^ < 50%), a fixed-effects model was used for the meta-analysis. If the heterogeneity between studies was high (*P* < .1, *I*^2^ ≥ 50%) we analyzed the reasons for the heterogeneity, and used the one-by-one elimination method to conduct sensitivity analysis to reduce the influence of heterogeneity. When there was statistical heterogeneity, but no clinical heterogeneity or a statistically significant difference between the 2 study groups, a random-effects model was used. Descriptive analysis was used when the heterogeneity of the other 2 groups was too large or the data source could not be found.

## 3. Results

### 3.1. Basic information of the included studies

**Figure 1.**
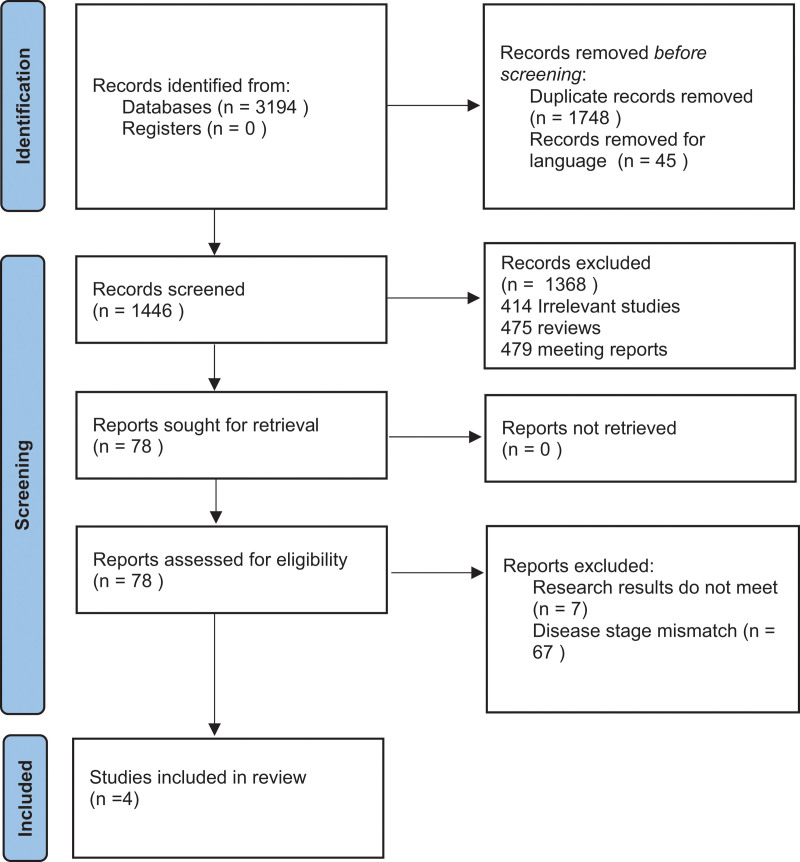
PRISMA flow diagram.

Seventeen studies met the relevant standards. The screening process is shown in Figure 1, and basic information on the included literature is shown in Table 1.

**Table 1:** Characteristics of included studies.

Author, year	Year of study	Research center	State	Group	Samples	Outcomes	NOS
Francesco Multinu, 2019^[13]^	2004–2013	Multicenter	USA	SLN	56	5-yr overall survival rate, chemotherapy rate, radiotherapy rate, lymphovascular invasion rate	8
				LND	48		
Giorgio Bogani, 2020^[14]^	2006–2016	Multicenter	Italy	SLN	40	5-yr overall survival rate	6
				LND	42		
Dimitrios Nasioudis, 2021^[15]^	2012–2015	Multicenter	USA	SLN	109	3-yr overall survival rate, chemotherapy rate, radiotherapy rate, lymphovascular invasion rate	8
				LND	1323		
Koji Matsuo, 2022^[16]^	2010–2018	Multicenter	USA	SLN	287	3-yr overall survival rate, chemotherapy rate, radiotherapy rate	9
				LND	5276		

LND = lymphadenectomy, NOS = New Castle-Ottawa Scale, SLN = sentinel lymph node.

### 3.2. Effects on survival rate

**Figure 2.**
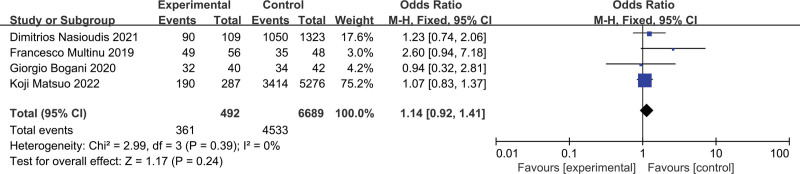
Meta-analyses of SLN group versus the LND group for total survival rate. LND = lymphadenectomy, SLN = sentinel lymph node.

**Figure 3.**
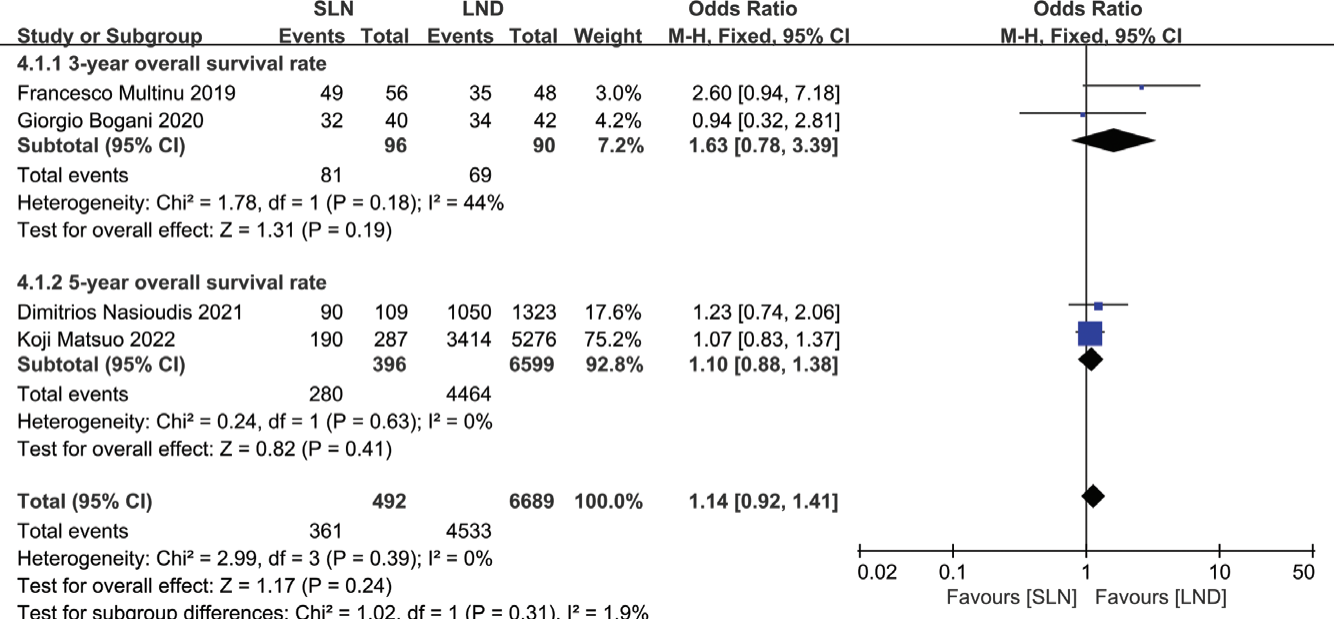
Subgroup analysis of SLN group versus the LND group for survival rate. LND = lymphadenectomy, SLN = sentinel lymph node.

Four studies were assessed, which included 7181 patients (SLN:LND = 492:6689). The results of the meta-analysis are shown in a forest plot (Fig. 2; *P* = .24 > .05), and the difference was not statistically significant. There was no statistical significance in the heterogeneity test (*P* = .39, *I*^2^ = 0%), indicating that the included studies were homogeneous, and the overall survival rates of the 2 groups were analyzed using a fixed effects model. The combined results showed that the OR = 1.14 (95% CI: 0.92–1.41); therefore, there was no statistical difference in the overall survival rate between the SLN group and the LND group. Moreover, the results of the subgroup analysis showed that there was no statistically significant difference between the SLN and LND groups in terms of the 3-year and 5-year overall survival rates (Fig. 3).

### 3.3. Effects of chemotherapy rate

**Figure 4.**
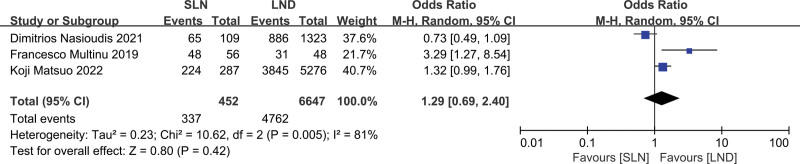
Meta-analyses of SLN group versus the LND group for chemotherapy rate. LND = lymphadenectomy, SLN = sentinel lymph node.

Three studies were assessed, which included 7099 patients (SLN:LND = 452:6647). The results of the meta-analysis are shown as a forest plot (Fig. 4; *P* = .42 > .05); the difference was not statistically significant. The heterogeneity test (*P* = .005, *I*^2^ = 81%) indicated that the included studies had high heterogeneity, which could not be reduced by the sensitivity analysis; therefore, a descriptive analysis was performed. Two studies suggested that the chemotherapy rates in the SLN and LND groups were similar, and 1 study showed that the chemotherapy rate in the SLN group was lower than that in the LND group.

### 3.4. Effects of radiotherapy rate

**Figure 5.**
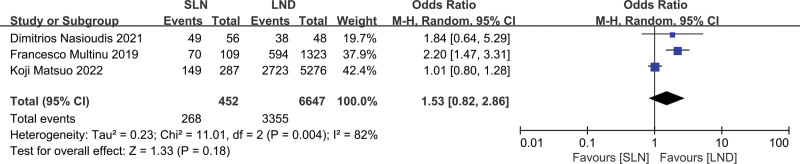
Meta-analyses of SLN group versus the LND group for radiotherapy rate. LND = lymphadenectomy, SLN = sentinel lymph node.

**Figure 6.**
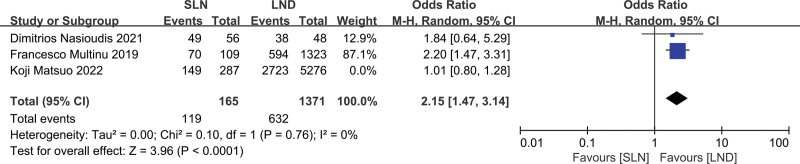
Meta-analyses of SLN group versus the LND group for radiotherapy rate (after exclusion of heterogeneity). LND = lymphadenectomy, SLN = sentinel lymph node.

Three studies were assessed, including 7099 patients (SLN:LND = 452:6647). The meta-results are shown in a forest plot (Fig. 5; *P* = .18 > .05), and the difference was not statistically significant. The heterogeneity test (*P* = .004, *I*^2^ = 82%) indicated that the included studies had high heterogeneity. After performing the sensitivity analysis, 1 study was eliminated to reduce the heterogeneity, and the remaining 2 studies were merged (Fig. 6). A further heterogeneity test (*P* = .76, *I*^2^ = 0%) indicated that the included studies were homogeneous. The difference between the 2 groups regarding the radiotherapy rate was significant (*P* = .0001 < .05), with a combined OR = 2.15 (95% CI: 1.47–3.14).

### 3.5. Effects on lymphovascular invasion rate

**Figure 7.**
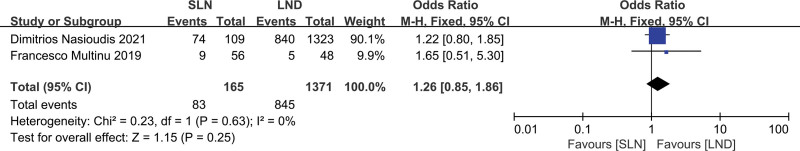
Meta-analyses of SLN group versus the LND group for lymphovascular invasion rate. LND = lymphadenectomy, SLN = sentinel lymph node.

Two studies were assessed, including 1536 patients (SLN:LND = 165:1371). The meta-results are shown in a forest plot (Fig. 7; *P* = .25 > 0.05), and the difference was not statistically significant. There was no statistically significant difference in the heterogeneity test (*P* = .63, *I*^2^ = 0%), indicating that the included studies were homogeneous. A fixed-effects model was used to analyze the lymphovascular invasion rates of the 2 groups. The combined results showed that the OR = 1.26 (95% CI: 0.85–1.86); therefore, it can be considered that there was no statistical difference in the lymphovascular invasion rate between the SLN group and the LND group.

## 4. Discussion

Lymph node metastasis is an important factor affecting postoperative adjuvant therapy and the prognosis of endometrial cancer. Previously, it was believed that LND could bring greater survival benefits to patients with endometrial cancer, which been used to establish extrauterine disease and as a therapeutic procedure. However, recent studies have found that, compared with no LND, systemic LND has little obvious survival benefit due to the enlargement of the scope of the operation and the increased incidence of postoperative complications, thereby reducing the quality of life of patients.^[7,17,18]^ Therefore, the necessity of LND remains controversial. However, it is difficult to assess lymph nodes that have metastasized without lymph node resection, and thus provide a reference for postoperative adjuvant therapy. Therefore, SLN assessment has emerged, which not only assessed the status of lymph nodes but also reduces the occurrence of postoperative complications.

SLNs are selective and limit the excision of tumor-specific or organ-specific lymph nodes and injection of a tracer into or around the primary tumor to identify and remove lymph node tissue that drains directly from the primary tumor site.^[19]^ The feasibility and accuracy of SLN assessment have been demonstrated in previous studies,^[20–22]^ which has also been included in the National Comprehensive Cancer Network guidelines as an option for staging surgery for endometrial cancer. SLNs identified with indocyanine green as a tracer have high accuracy in detecting endometrial cancer metastasis and can replace LND for surgical staging of endometrial cancer.^[23]^

The main purpose of SLN biopsy is to identify the lymph nodes most at risk of metastasis and to reduce the morbidity of associated complications by limiting complete LND.^[24]^ Although SLNs are widely used as alternatives for low-risk endometrial cancer, their use in high-risk endometrial cancer remains controversial. However, SLNs may reduce the need for standard LND and do not appear to adversely affect the detection of stage IIIC disease,^[24]^ which provides a reference for the use of SLNs in high-risk cases. Simultaneously, with the increasing use of SLNs, the incidence has also gradually increased in patients with extrauterine involvement.

Cytoreductive surgery is the mainstay of surgery for patients with advanced endometrial cancer. However, LND appears to have no apparent survival benefit and is associated with an increased risk of complications. Therefore, role of LND in the treatment of tumors remains controversial due to it being a local treatment that is more likely to improve locoregional control and less likely to affect systemic disease. The effect of LND may not be sufficient to treat lymphatic spread, and there is no apparent survival benefit from LND without effective systemic adjuvant therapy.^[25,26]^ Under normal circumstances, most patients with suspected pelvic lymph node metastases are treated with adjuvant postoperative consolidation therapy. If only lymph node metastasis is identified, the dissection of the pelvic lymph nodes is enough.^[27,28]^ Therefore, SLN resection may be an alternative to comprehensive LND as a treatment option for advanced endometrial cancer.

Four retrospective studies were included in this meta-analysis. In terms of survival rate, the results showed that there was no significant difference in the overall survival rate between the SLN group and the LND group (OR = 1.14, 95% CI: 0.92–1.41, *I*^2^ = 0%, *P* = .39), that is, LND in patients with advanced endometrial cancer did not significantly improve the survival prognosis compared with SLN assessment. The subgroup analysis also showed that there was no statistically significant difference in the 3-year overall survival rate or the 5-year survival rate between the SLN and LND groups, which may imply that the impact on the short-term, or even long-term, prognosis may not be different. Moreover, the routine performance of LND adds to the operation time, costs, intraoperative bleeding, and complications, such as lower extremity lymphedema, lymphocysts, and hydronephrosis, which seriously affect the quality of life of patients after surgery.^[6,29]^ Therefore, SLN assessment is more advantageous than LND for improving the patient quality of life.

The most important aspect of lymph node evaluation is to guide postoperative adjuvant therapy. In this study, the lymphovascular invasion rate in the 2 groups was not significantly different (*P* = .25). In terms of postoperative adjuvant therapy, the rates of postoperative chemotherapy and radiotherapy were included in 3 studies. The results showed that there was no significant difference in the postoperative chemotherapy between the 2 groups (*P* = .42); however, due to the high heterogeneity, there may have been some differences in chemotherapy rates according to the descriptive analysis. In terms of postoperative radiotherapy, the results showed a statistically significant difference (*P* = .001); the SLN group had a higher chemotherapy rate than the LND group, which may have an impact on the prognosis of patients with advanced endometrial cancer. However, owing to the small number of included studies, more statistical analyses of the literature and related case data are needed.

An individual patient condition and actual situation are different; therefore, it is impossible for every patient to have the same diagnosis and treatment plan. According to a research analysis, the hospitalization time of patients with a body mass index ≥ 40 kg/m^2^ is prolonged, and the incidence of postoperative wound infection and venous thrombophlebitis are increased. Furthermore, obese patients have a higher risk of complications, and minimally invasive surgery helps to prevent overall complications. Therefore, the fragility index assessment can identify high-risk patients, prevent and diagnose complications as early as possible, help clinicians to intervene in time, and strives for better conditions for patient follow-up treatment.^[30]^ However, patients with poor basic conditions, especially elderly women, may be at a higher risk for surgery when complicated by basic diseases; therefore, SLNs could be used instead of systematic LND to evaluate the lymph nodes in these patients to reduce the risk of postoperative complications. This also shows that, based on the guidelines and the principles of diagnosis and treatment, we should make the most suitable diagnosis and treatment plan for patients according to the actual situation of each patient and combine it with our own experience to achieve an individualized treatment plan.

With the progress in cancer genetics and molecular biology, targeted drug therapy is also accelerating. In 2013, based on the genomic characteristics of endometrial cancer, The Cancer Genome Atlas identified 4 distinct subgroups: POLE ultramutated, microsatellite instability hypermutated, copy number-low, and copy number-high. Each molecular subtype has a different prognosis and plays an important role in guiding individualized treatment.^[31]^ An increasing number of studies have shown that the molecular subtype is more accurate than traditional histological features for evaluating patient risk and further adjuvant treatment. Choosing appropriate biomarkers can improve the long-term survival rates of patients.^[32]^

Preoperative genomic testing to understand molecular subtypes can also be used to guide surgery and adjuvant therapy, which may reduce the risk and cost of surgery. Endometrial cancer with ultramutated POLE has been proven to have a good survival rate, whereas the prognosis of copy number-high POLE is relatively poor. Diagnostic endometrial biopsy or curettage specimens can be used for genomic testing with high accuracy, consistent with the results of hysterectomy specimens. It can guide surgical methods for patients who want to preserve fertility or reduce the risks related to surgery.^[33,34]^ Some studies have found that the molecular subtype of endometrial cancer is significantly related to lymph node metastasis, and that lymph node evaluation may not be beneficial for patients with ultramutated POLE.^[35]^ This indicates that the subtypes with a good prognosis can be treated without lymph node assessment, or only SLN assessment, which requires further study.

Presently, clinical research and trials are focusing on targeted therapy, and molecular and genomic profiling provides a practical treatment plan for endometrial cancer. However, the high cost and long turnaround time of molecular and genomic testing have become the main obstacles for molecular typing. Some studies have suggested that evaluation of radiomics and radiogenomic signatures may provide results similar to those of molecular testing and could become an important tool for an accurate diagnosis and better individualized treatment. In view of this, it is necessary to formulate more complex prognostic scores involving a growing number of gene alterations so that we can make accurate diagnoses and formulate individualized treatment plans using radiomic features instead of molecular or genomic profiling.^[36]^

The purpose of the present meta-analysis was to evaluate the prognostic impact of SLN assessment compared with LND in patients with advanced endometrial cancer. Overall, this study suggests that there may be differences in postoperative adjuvant therapy; however, these may not affect patient survival after surgery. Since most of the literature on SLNs in endometrial cancer is retrospective, with different sample size and outcome indicators, as well as ethical issues, it is better apply SLN assessment in clinical practice; therefore, more randomized controlled trials and large-scale studies are required for further evaluation.

## 5. Conclusion

SLN biopsy may be an alternative to LND for the treatment of advanced endometrial cancer. Based on the guidelines and principles of diagnosis and treatment, we should choose the most appropriate treatment plan according to the specific situation of patients and evaluation method of the prognosis.

## Acknowledgment

We would like to thank Editage (www.editage.cn) for English language editing.

## Author contributions

**Conceptualization:** Huiyi Yao.

**Data curation:** Huiyi Yao, Ruiwen Luo, Ruoyi Tong.

**Formal analysis:** Yanwen Wei.

**Methodology:** Yanwen Wei.

**Software:** Kaiteng Zheng.

**Validation:** Xiangdan Hu.

**Writing – original draft:** Huiyi Yao.

**Writing – review & editing:** Xiangdan Hu.
